# Nursing interventions in monitoring the adolescent with Cystic Fibrosis: a literature review

**DOI:** 10.1590/1518-8345.1396.2845

**Published:** 2016-12-08

**Authors:** Maria da Conceição Marinho Sousa Ribeiro Oliveira Reisinho, Bárbara Pereira Gomes

**Affiliations:** 1 Doctoral student, Instituto de Ciências Biomédicas Abel Salazar, Porto, Portugal. Adjunct Professor, Escola Superior de Enfermagem do Porto, Porto, Portugal.; 2 PhD, Professor, Escola Superior de Enfermagem do Porto, Porto, Portugal.

**Keywords:** Nursing Care, Adolescents, Cystic Fibrosis, Quality of Life, Self-Care

## Abstract

**Objectives:**

to search for nursing interventions focused on the improvement of quality of life and promotion of self-care of adolescents suffering from the Cystic Fibrosis.

**Method:**

literature review. The inclusion criteria were: primary studies and studies with interventions developed by nurses in the adolescent population with Cystic Fibrosis, using Portuguese, Spanish, French and English with no time limit, and supported by the databases Scopus, Web of Science and CINAHL. The search expressions were: nursing AND care AND adolescent AND "Cystic Fibrosis" AND ("quality of life" OR "self-care").

**Results:**

a total of 59 articles was retrieved; 8 matched the criteria chosen. Nursing interventions targeted at adolescents with Cystic Fibrosis and their family members were identified. These interventions were organized according to the nurses' role, namely caregiver, coordinator, counsellor, researcher, trainer and care partner.

**Conclusions:**

nursing interventions targeted at following up the adolescent during the entire therapeutic process, involving the presence of parents/significant others, since both the adolescent and family have to be responsible for self-care. Healthcare professionals should be capable of identifying the specific needs of patients with chronic disease and their family, permitting a better understanding and adaptation to the health-disease transition process.

## Introduction

Adolescence is characterised by major fluctuations, particularly in physical terms - of rapid and accelerated growth until maturity; at a cognitive level - from the abstract to the fully established capacity for abstract thinking; in terms of identity - where the body image causes a variety of concerns until it becomes clearly defined; regarding relations with parents - from the definition of boundaries, through major conflicts until the achievement of emotional and physical separation; in terms of relationships with peer groups - search for the acceptance of friends, fear of rejection, to an interest in individual friendship to the detriment of the group; sexuality - fluctuating between self-exploration and the formation of stable relationships with others; and major mood swings until the establishment of greater emotional stability in terms of mental health^1^. If adolescence may be described as a troubled life cycle, it will become even more difficult if the adolescent has to deal with a chronic disease. Nurses are members of a healthcare team that takes care of adolescents with Cystic Fibrosis. Each member of the multidisciplinary team has a specific role; hence, the nurse's work should be guided by an approach that emphasises the development of practice. Nurses can help chronic patients to control the repercussions of their disease since, at the present date, a cure is almost if not truly impossible. Thus, the nurse's focus of attention should be the patient, namely the adolescents, as the control centre of the actual disease, i.e. to support the patient, in the training of his/her capacity to self-manage the disease through effective and individual projects. 

This research aimed to search for scientific evidence to guide nursing clinical practice. The choice of Cystic Fibrosis and the selection of adolescence as the age group are related to the fact that this is a rather unknown condition among the public in general and a disease that shows the strongest clinical expression in children and adolescents. Cystic Fibrosis, also called Cystic Fibrosis of the Pancreas and Mucoviscidosis, is a chronic, genetic, hereditary disease, most frequent in Caucasians. Cystic Fibrosis is transmitted in a recessive autosomal form, meaning that this disease is passed on by both parents of the child. Various authors refer to an incidence that fluctuates between 1:2000 and 1:1500 of newborn infants in the European population. It is less frequent in Africans and rare in Asians^2^ ^-^ ^3^. Cystic Fibrosis affects various organs and is characterised by the dysfunction of the exocrine glands. The secretions are very thick, due to alteration in the functioning of exchanges of water and salt in the exocrine gland cells. These secretions will cause obstruction in various organs, and manifest themselves in the lungs, pancreas, intestines, reproductive system and sweat glands. Therefore, patients can present various clinical manifestations, isolated or together in relation to the affected organ: chronic cough, recurrent pneumonia, low weight, deficient food absorption, pancreatitis, meconium ileus and elevated sweat chloride^2^ ^-^ ^3^. Increased research, namely on specific treatments to the several mutations, as well as the creation of specific Cystic Fibrosis treatment centres (health units where adolescents are monitored by several health professionals from the multidisciplinary team - physician, nurse, physiotherapist, psychologist, nutritionist - thus enhancing the quality of care provision) has helped to improve the quality of life of adolescents and increase their life expectancy. Hence, in conducting this research, it is hoped that data may be found upon which to formulate guidelines, helping to mitigate the daily problems for those carrying this disease and their family.

The contact of the nurse with adolescents with Cystic Fibrosis becomes rather diversified due to changes in their growth and development, and is also related to the limitations of the chronic disease which accompany adolescents in their daily life. Caring for adolescents with a chronic disease implies knowing the different circumstances of these patients' daily lives, whether these are factors related to the actual disease, such as family factors (family atmosphere, household members, socioeconomic status, degree of interference in family organisation), personal factors (temperament, motivation, problem-solving capacity, cognitive and intellectual capacity, self-awareness and self-esteem) and socio-environmental factors (social support and support from group of friends, community resources and school^4^ ^-^ ^5^. The parents and family of these adolescents also need attention, as there are interconnections between the different members of the family. The role of the family in the wellbeing of the adolescent is a determining factor in the adolescent's capacity to adapt to this chronic disease. Emotional reactions, family functioning, parental function, special concerns and needs are areas identified as potentially able to cause disorders associated to everyday parental/family experiences of chronic disease in children/adolescents^5^.

Healthcare professionals should have detailed information to be able to identify the different phases of the chronic disease and the way that adolescents and their parents/family deal with the situation. According to one study it is "indispensable that changes occur in the attitudes of professionals in the daily provision of assistance and in training, so as to equip these professionals with the capacity required for care of chronic conditions in adolescence"^6^. If the practice of nursing is based on evidence, the coordinated care provided to carriers of the chronic disease can become more effective and encompassing, and thus enable an understanding of the implications of the disease for the family, the psychosocial effects on adolescents and members of their family, the issues of triage and transition of care and the form of assistance given to these adolescents and families.

The nurses play a decisive role in the follow-up provided in the different phases of life and in the different phases of the disease, and should also be the coordinating element of the healthcare team that takes care of these adolescents and their families^6^ ^-^ ^7^. In Portugal, nurses specialising in Cystic Fibrosis simply do not exist; hence, children and adolescents are looked after by nurses providing general care, rehabilitation nurses or child health and paediatric specialists. However, these are nurses who possess knowledge in this particular area. The College of the Speciality of Child Health and Paediatrics of the Portuguese Nurses' Association, recommends that a nurse should "work in partnership with the adolescent and family/significant person, in any context in which the adolescent is found (hospitals, continuous care, health centres, school, community, home, ...), so as to promote the highest status of health possible, provide care to a healthy or sick adolescent and provide education towards health as well as identify and mobilise resources to support the family/significant person"^8^.

It has become necessary to carry out research on published scientific articles, which identify nursing interventions that improve the quality of life and self-care of the adolescent with Cystic Fibrosis.

## Method

Literature review. We embarked on this research with the following initial question: which nursing care directed at adolescents with Cystic Fibrosis improves the quality of their life and boosts their self-care?

**Figure 1:**
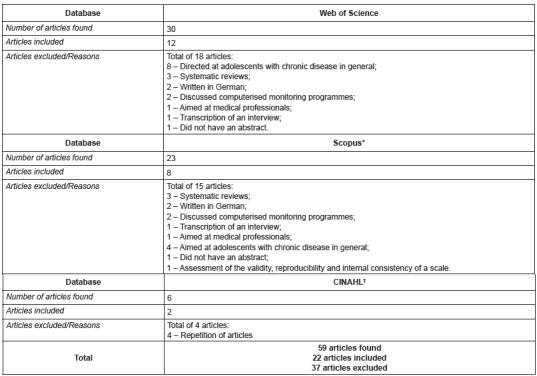
Databases and selection of articles

The active search for publications was carried out in July 2015 in the following databases: Scopus (www.scopus.com), Web of Science (www. isiknowledge.com) and CINAHL (http://search.ebscohost.com). The above databases and indexes were selected given their wide content scope in the health sciences domain. The data search in CINAHL is mandatory for articles in the nursing field. In addition, the Scopus and Web of Science databases permitted a cross-reference search with indices, namely Medline, EMBASE, Cochrane Database of Systematic Reviews, Social Sciences Citation Index, Science Citation Index and Conference Proceedings Citation Index, data sets of major importance for this type of study. The search terms used were nursing AND care AND adolescent AND "Cystic Fibrosis" AND ("quality of life" OR "self-care"), with the following inclusion criteria: primary studies and studies with interventions developed by nurses, the population being adolescents with Cystic Fibrosis, written in Portuguese, Spanish, French and English languages and no time limit. The articles that did not include the full text were excluded from the final analysis. Figure 1 shows the articles extracted from the selected databases.

* Reference Database; † Cumulative Index to Nursing and Allied Health Literature

From the 59 extracted articles, 37 were excluded for not meeting any of the inclusion criteria and 22 were included after considering the title and abstract. However, eight repeated articles were found in the three databases, thus resulting in 14 final articles for analysis. From these 14 articles, it was not possible to have full access to 6 articles, which was necessary for a complete analysis. Later attempts were made to contact these authors but one had a wrong email address, three articles required payment for full access, one author was already deceased and one did not reply.

**Figure 2:**
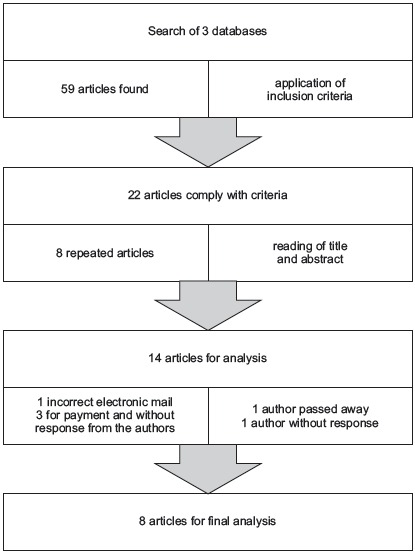
Summary of the search

In Figure 2, the article selection process is summarized. 

In order to identify the nursing interventions provided to adolescents with Cystic Fibrosis, eight articles were analysed with respect to: study objectives, research design, results (nursing interventions) and conclusions.

The articles were assessed by another researcher, independently, and subsequently classified according to: "type of study"; "objectives", "nursing interventions" and "main conclusions".

## Results

The 8 articles included in this review show different research methods: two studies are descriptive/reflexive; one is exploratory/descriptive; one is a case study; one is a survey; one is correlational; one is a content analysis and one is a qualitative update. The analysis of the articles relative to the study design highlights various particularly salient aspects, such as their qualitative method and objectives, which refer to the area of knowledge and understanding (description, understanding, reflection and exploration).

Nursing interventions targeting adolescents with Cystic Fibrosis and their family members were identified. These interventions will be displayed and organized according to the nurses' role, namely caregiver, coordinator, counsellor, researcher, trainer, and care partner.

**Figure 3:**
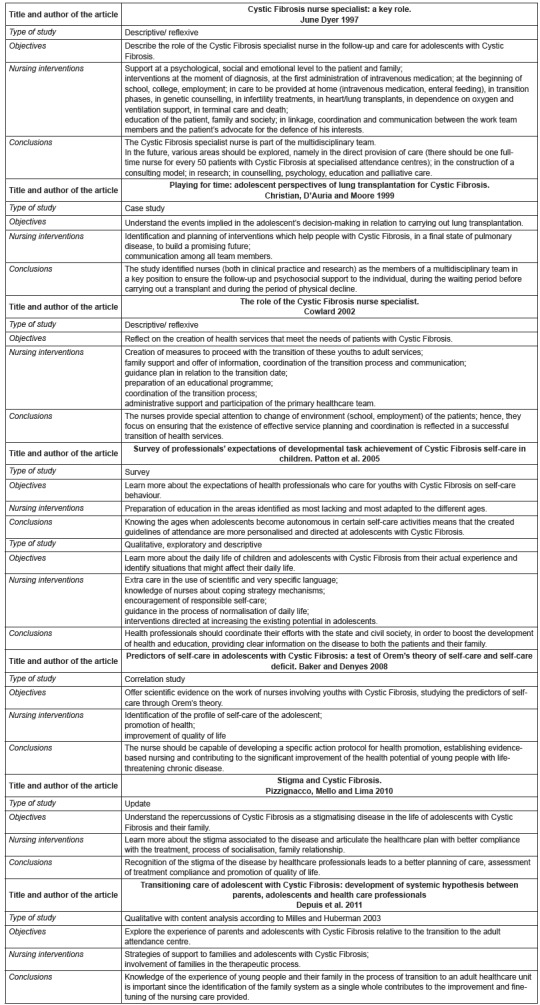
Information obtained from the analysis of selected articles

Figure 3 condenses all the information obtained from the eight articles that comply with all the criteria defined *a priori.* The figure shows the article's author identification, as well as the research method applied, the proposed goals and the identification of nursing interventions, which was the basis of the research.

## Discussion

This study revealed that nurses plan and implement interventions targeted at the adolescents' needs in the several stages of life and disease, and also provide support to family caregivers, namely the parents.

Caring for an adolescent with a chronic disease, such as cystic fibrosis, involves a multidisciplinary work and this is also one of the nurses´ responsibilities. This professional is responsible for interacting with community services and governmental institutions, aiming to provide the best quality care to these patients, namely in what concerns pre and post pulmonary transplantation^9^ ^-^ ^11^.

Results also show clear evidence on the nurse's role as a caregiver, particularly in what concerns inhaled and intravenous medication administration, enteral and parenteral nutrition, ventilator weaning and oxygen administration^9^. However, in addition to these nursing interventions, related to the know-how, the educational domain is also highlighted by several authors as an important competence of nurses who provide support to patients and also to their family caregivers, thus contributing to extended home care^9^ ^-^ ^12^. 

In this literature review, all studies refer to nursing interventions, mainly focused on communication processes, considered an important and efficient method to provide emotional and psychologic support, aiming at tailor-made care plans.

Encouraging the involvement of family members in the therapeutic process and the support to the family as the most important promoter of the adolescent well-being is a key factor that will enable the adolescent to better adapt to the chronic disease^12^.

The identification of educational needs is crucial for nursing, especially at the time of diagnosis, in genetic counselling, in the life cycle transition periods in pre and post pulmonary transplantation, hospitalization and return home, in daily life activities, re-entry in school and in the promotion of the quality of life of adolescents with cystic fibrosis and family caregivers^9^ ^,^ ^11^ ^-^ ^13^.

Emphasis should also be given to the nurses' role in training these adolescents and their family members to pay special attention to feeding, nebulization, oxygen therapy, which will most likely result in benefits to the adolescent and avoid hospital readmissions^9^.

The nurse, as a health team member who interacts closely with the adolescent and family in the transition health-illness process, is able to establish a close relationship and offer emotional support, contributing to the autonomy of the adolescents and their family members^9^ ^,^ ^11^ ^-^ ^12^ ^,^ ^14^ ^-^ ^15^.

Developing the potential of each individual with cystic fibrosis and advocate for the patient's legal interests and protection is also one of the nurses' competencies^(9- 11)^.

The partnership of care and the identification of critical areas, such as the stigma associated with this disease, contribute to improve treatment compliance and enhance quality of life^16^.

Many authors study the transition to adult health services, since the developments in care for adolescents and training provided to families has helped to increase these patients' life expectancy, thus becoming a new area of knowledge to be explored^13^ ^,^ ^15^. Authors have reflected on the need to implement measures to help these adolescents' transition to adult health services, as well as the focus areas that nurses should consider, namely the identification of the family support and information provided, coordination of the transition and information process^13^ ^,^ ^15^.

The evidenced-based research and practice developed by nurses empowers them with the necessary skills to enable a better health promotion and support the development of guidelines underlying nursing practice^10^ ^,^ ^14^.

The provision and management of care, the early identification of the adolescents´ needs, the interpersonal and therapeutic relationship established between nurses and adolescents/ families are important skills of nurses who provide care and support to this population. These are specific interventions, comprising specific areas such as primary, secondary and tertiary care, and are targeted at patients and family members from the moment of the disease diagnosis until the patients´ death^9^ ^-^ ^15^.

## Conclusion

Due to the scarcity of studies published by nurses in Portugal, it became necessary to conduct a search of articles to identify nursing care provided to adolescents with Cystic Fibrosis and thereby learn about the situation of nurses from other countries. Our review revealed nursing interventions directed at following up the adolescent during the entire therapeutic process without neglecting the presence of parents/significant family, since the procedure for making adolescents accountable for their self-care should always be the same as for their parents.

Healthcare professionals should be capable of identifying the particular needs of patients with chronic disease and their family, permitting the understanding of and adaptation to the health-disease process.

The importance of the nurse as an active member in the multidisciplinary team accompanying these patients was stressed by all the authors examined, which again reinforces the indispensable role of the nurse as a healthcare professional.

If the practice of nursing were evidence-based, the coordinated care provided to patients with Cystic Fibrosis could become more effective and comprehensive, and thus include the implications of the disease on the family, the psychosocial effects on adolescents and family members, issues of screening and transition of care and the form of assistance provided to these adolescents and families.
